# Supplementary material for: MetaRibo-Seq measures translation in microbiomes
Source: Nat Commun. 2020 Jun 29;11:3268. doi: 10.1038/s41467-020-17081-z (PMC7324362; doi:10.1038/s41467-020-17081-z)
Supplement: Supplementary file 10 — Supplementary Data 7 [file 41467_2020_17081_MOESM10_ESM.zip › File2/Confidence_High_Taxonomy/440057_out.krona.html]

Javascript must be enabled to view this page.

members
magnitude
magnitudeUnassigned
count
unassigned
taxon
rank

440057\_out

8

superkingdom
2
8

8
phylum
1239

class
186801
8

8
order
186802

3
family
541000

genus
1263
3

3

SRS049959\_contig\_number\_28014SRS144537\_contig\_number\_38585SRS148721\_contig\_number\_36213
species
2293234

family
186803
5

5
572511
genus


SRS015431\_contig\_number\_contig-100\_272.483553SRS016095\_contig\_number\_30600SRS018351\_contig\_number\_18196SRS062654\_contig\_number\_12342SRS104636\_contig\_number\_15270
5
33039
species
